# Dexamethasone therapy in adolescents with inadequately controlled congenital adrenal hyperplasia: effects on hormonal Suppression, Puberty, and gonadal outcomes

**DOI:** 10.1007/s12020-026-04577-9

**Published:** 2026-03-10

**Authors:** N. Berna Çelik Ertaş, Burcu Şenkalfa, Doğuş Vurallı, Z. Alev Özön, E. Nazlı Gönç

**Affiliations:** Department of Pediatrics, Division of Pediatric Endocrinology, Hacettepe University, Ankara, Turkey

**Keywords:** Adolescents, Congenital adrenal hyperplasia, Dexamethasone, Gonadal functions

## Abstract

**Purpose:**

Puberty poses challenges for the management of congenital adrenal hyperplasia (CAH) due to changes in the endocrine milieu and non-compliance with treatment. Uncontrolled hyperandrogenemia is a significant problem, especially for CAH patients in this age group. Along with the attainment of final height, a therapeutic window emerges for employing more potent glucocorticoids (GCs) to improve disease management in the pediatric age group. Dexamethasone (Dex) is a long-acting and more potent GC, and offers a convenient dosing schedule for patients. In this study, we aimed to evaluate the efficacy of Dex treatment on biochemical and clinical hyperandrogenemia in patients with inadequately controlled CAH.

**Methods:**

Children with CAH who had persistent biochemical/clinical hyperandrogenemia despite recommended hydrocortisone dosing were included in the study at a single institution within the past 20 years. Participants were switched to Dex therapy at or near final height. Clinical and laboratory data and treatment outcomes for all participants were retrospectively evaluated.

**Results:**

Thirty-two patients (karyotype 46,XX in 18/32, 46,XY in 14/32) with a mean age of 15.5 ± 2 years were included in the study. 3 of 46,XX cases were male-reared (M^R^), and so a total of 17/32 (53%) of the patients were M^R^, and 15/32 (47%) were female-reared (F^R^). Following the transition to Dex therapy, both 17-hydroxyprogesterone (17OHP) (*p* < 0.01) and 1,4-delta-androstenedione (A4) (*p* < 0.01) levels showed a significant decrease after 6–12 months in all patients. In F^R^ patients, puberty progressed in 5/5, menstrual regularity improved in 3/5, and hirsutism improved in 3/7. In M^R^ patients, A4/testosterone ratio decreased in 10/10, testis volumes increased in 7/8. While there was no change in BMI z-score during treatment in M^R^ patients, an increase in BMI z-score was observed only during the first six months in F^R^ patients (*p* = 0.014).

**Conclusion:**

Dex effectively suppresses adrenal androgens and can be used to restore the hypothalamo-pituitary-gonad axis in children with CAH who have uncontrolled hyperandrogenemia and have reached final height. To minimize weight gain and metabolic side effects, treatment should be initiated at a low dose and androgen levels should be monitored without delay.

## Introduction

Congenital adrenal hyperplasia (CAH) is caused by various enzymatic defects within the adrenal steroidogenesis. Treating CAH involves carefully balancing hypercortisolism and hyperandrogenemia, often posing challenges for endocrinologists. Different glucocorticoid (GC) treatment regimens, including hydrocortisone (HC), prednisone, prednisolone, and dexamethasone (Dex), or combined GC preparations, administered in a circadian or reverse circadian manner, could be utilized to achieve normal adrenal androgen levels [1]. However, it might not always be possible to get the desired target levels of adrenal androgens. Finkielstain et al. reported a cohort of 170 children with CAH, of whom only 50% had normal 1,4-delta-androstenedione (A4) levels [2]. Additionally, puberty poses challenges for the achievement of adequate control [3, 4]. Puberty-induced changes in the endocrine milieu impact the pharmacokinetics of HC, which worsens therapeutic management [5–9]. Psychosocial factors related to puberty may lead to non-compliance with treatment and exacerbate the challenges associated with physiological alterations in the endocrine milieu [10]. Due to the impact of long-term hyperandrogenism, children and adolescents with CAH are more likely to develop cardiovascular disease in adulthood [11, 12]. In addition, inadequate control of disease also leads to the development of gonadal rest tumors, secondary hypogonadism, and decreased fertility [12].

At the end of puberty, as concerns about final height diminish, interventions aimed at improving gonadal function and metabolic regulation become more important in CAH management. Consequently, a therapeutic window emerges for employing more potent GCs to improve disease management in patients with CAH. Dex is a long-acting GC preparation. The half-life of Dex allows for once- or twice-daily usage, offering a more convenient dosing schedule for patients. In patients with CAH, Dex demonstrates superior efficacy in suppressing adrenal androgens compared to HC [13–17]. In limited case reports and case series of adolescent patients with poor hormonal control, Dex therapy was associated with normalization of adrenal androgens and the onset and regularity of menstrual cycles [18–20]. However, these reports did not focus on pubertal progression and gonadal function, especially in male adolescents with CAH. In this retrospective study, we evaluated the efficacy of Dex treatment regimen in patients with CAH who attained final or near-final height, where HC treatment was inadequate in controlling hyperandrogenemia. We focused on pubertal development, menstrual regularity, and the presence of testicular or ovarian rest tumors in patients with CAH who switched from HC to Dex.

## Material and methods

Thirty-two patients with classic CAH who were followed during a 24-year period (2000–2024) at a university hospital setting were reviewed retrospectively. Patients aged under 18 years old, diagnosed with 21-hydroxylase (salt wasting (SW) or simple virilizing (SV)), and 11-beta-hydroxylase deficiencies, and attained final or near-final height were included. The near-final height was defined as a bone age of ≥ 14 years in females and ≥ 16 years in males, an annual growth rate of less than 2 cm. The final height was defined as the point when the growth plates were fully closed.

Despite administering the recommended HC dose (15–20 mg/m²/day, at least three times daily), patients with persistent biochemical and clinical hyperandrogenemia that lasted for at least 12 months or across three clinical visits were considered to have ‘inadequate disease control’, and the treatment was switched to Dex [21]. Biochemical hyperandrogenemia was defined by A4 levels more than 1.5 times the upper normal limit and/or morning pre-dose 17-hydroxyprogesterone (17OHP) above 10 ng/mL [22, 23]. Hirsutism, delayed puberty, menstrual irregularities, testicular and ovarianadrenal rest tissue (TART, OART), or its progression denote clinical hyperandrogenemia. The initial dosage of Dex was established as 15 mg/m^2^/day of HC equivalent doses (assuming 1 mg Dex is equivalent to 40 mg HC) once daily [24]. The dose was adjusted every 1–3 months based on adrenal androgen levels, with doses increasing or decreasing by 25%, targeting the age and gender appropriate adrenal androgen levels. The cumulative Dex dosage was calculated as the sum of daily Dex doses, expressed as HC equivalent doses of Dex, starting from the treatment switch and continuing until the next available measurement of weight and height. Fludrocortisone treatments were continued in SW-CAH patients (0.025-0.1 mg/day).

Patients were categorized based on the sex of rearing. Patients with 46,XY karyotype and 46,XX patients reared as males were labeled as male-reared (M^R^). The remaining patients with 46,XX karyotype were labeled as female-reared (F^R^).

All clinical and laboratory data were obtained from medical records. Puberty was staged according to Tanner (Tanner breast, T_B_ for females; Tanner genital, T_G_ for males) [25]. Delayed puberty was defined as the absence of testicular enlargement in boys and breast development in girls at the ages of 14 and 13 years, respectively [26]. Clinical and laboratory (gonadotropins, sex steroids) evaluation of puberty was not carried out in M^R^ patients with a 46,XX karyotype (3 patients). Weight, height, and body mass index (BMI; kg/m^2^) standard deviation scores (SDS) were assessed using the Centers for Disease Control and Prevention (CDC) charts [27]. The change in BMI z score over a one-year period at three time points (baseline, 6th, and 12th month) of treatment was analyzed. Percentiles for systolic and diastolic blood pressures (SBP and DBP) were determined based on age, gender, and height [28]. Menstrual pattern (normal, oligomenorrhea, metrorrhagia, and primary and secondary amenorrhea) was noted [26]. Six patients had menarche, one of whom developed hematocolpos, thus excluded from the analysis, and three patients had primary amenorrhea [29]. The remaining six patients did not fulfill the criteria for menstrual evaluation; consequently, eight F^R^ patients were evaluated for menstrual pattern. The presence of hirsutism in females was recorded. A Ferriman-Gallwey score of 8 or higher was assumed to indicate hirsutism [30]. Plasma adrenocorticotropic hormone (ACTH) (pg/mL), adrenal androgens (17OHP (ng/mL), A4 (ng/mL), 11-deoxycortisol (11DOC) (ng/mL)), gonadotropins (luteinizing hormone (LH) (mIU/mL), follicular stimulating hormone (FSH) (mIU/mL)), and sex steroids (estradiol (E2) (pg/mL), testosterone (T) (ng/dL)), serum sodium and potassium (mEq/L), plasma renin (pg/mL), and plasma glucose levels (mg/dL) were all noted. Adrenal androgens were measured between 08.00 and 09.00 a.m., prior to the administration of the morning dose of GCs. The A4 to T ratio (A4/T) was used to differentiate testicular vs. adrenal T production for males (after converting all values to the same units) [31]. An A4/T ratio < 0.2 indicated T from testicular origin, while a ratio > 1 suggested T predominantly from adrenal origin in males. An A4/T ratio between 0.2 and 1 indicated mixed adrenal and testicular production [31]. The A4/T ratio was calculated in M^R^ patients with a 46,XY karyotype, whose A4 and T levels were measured simultaneously. Therefore, the ratio was calculated in 10 M^R^ patients. M^R^ patients with a 46,XX karyotype were included only in 17OHP and A4 evaluations. The presence of TART or OART on ultrasonography was recorded. The presence of Cushingoid features, such as a rapid increase in weight, a moon face, a buffalo hump, purple or bright red striae, and/or hypertension following the treatment switch, was recorded.

FSH, LH, E2 (ARCHITECT System, Abbott Laboratory Diagnostics, USA), and T (IMMULITE 2000 System, Siemens, UK) levels were measured using an immunochemiluminometric assay (ICMA). The lowest measurable levels of FSH, LH, E2, and T were 0.3 IU/L, 0.3 IU/L, 10 pg/mL, and 20 ng/dL, respectively. Serum 17OHP level was measured by radioimmunoassay (RIA) (DIAsource ImmunoAssays, Belgium) with a lowest limit of detection of 0.09 ng/mL, intra- and interassay CVs of 4.6–10.7% and 7.6–19.2%, respectively. Serum 11DOC level was measured by RIA (DIAsource ImmunoAssays, Belgium) with a lowest limit of detection of 0.11 ng/mL. Plasma ACTH and serum A4 were measured using IMMULITE 2000 System using the ICMA method. Plasma ACTH measurement has a sensitivity of 5 pg/mL, intra- and interassay CVs of 2.5% and 3.6% respectively. Plasma renin was measured using immunoradiometric assay (DIAsource ImmunoAssays, Belgium) with a lowest limit of detection of 0.78 pg/mL, intra- and interassay CVs of 3.0-8.5% and 4–11%, respectively.

The study protocol was approved by the local ethics committee (Approval Number: 2024/14–25, Project Number: SBA 24/887). Informed consent was waived due to the retrospective nature of the study.

### Statistical analysis

All statistical analyses were carried out using SPSS 21.0 for the Windows software package (IBM Corp. Armonk, NY, USA). Normality was tested using the Shapiro-Wilk test. Descriptive analyses were presented using mean and standard deviation for normally distributed data, median, and interquartile range (IQR) for non-normally distributed data. Mean values of continuous variables were compared using t-tests; medians were compared using the Mann-Whitney U test. Paired samples t-test / Wilcoxon signed rank test or McNemar test were used for paired continuous or nominal data, respectively. A p-value < 0.05 was considered to be statistically significant.

## Results

Thirty-two patients with a mean age of 15.5 ± 2 years were included in the study. 27 patients had 21-hydroxylase deficiency (21/27 SW, 6/27 SV), and 5 patients had 11-beta-hydroxylase deficiency (Table 1).

**Table 1:** Baseline characteristics of the patients BMI: body mass index, F^R^: female-reared, M^R^: male-reared

		*n*	F^R^ (*n* = 15)	*n*	M^R^ (*n* = 17)
Diagnosis, *n* (%)	21-hydroxylase, salt wasting 21-hydroxylase, simple virilizing 11-beta hydroxylase	15	9 (60) 5 (33.3) 1 (6.7)	17	12 (70.5) 1 (6) 4 (23.5)
**Karyotype**,** n (%)**	46,XX 46,XY	15	15 (100) 0	17	3 (17.7) 14 (82.3)
**Age (years) (mean ± SD)**		15	15.2 ± 2.3	17	15.7 ± 1.7
**Height z score (mean ± SD)**		15	-0.8 ± 0.9	17	-0.5 ± 1.2
**BMI z score (mean ± SD)**		15	1.4 ± 0.8	17	1.05 ± 1.1

Karyotype analysis revealed 46,XX in 18 patients (18/32, 56%), and 46,XY in 14 patients (14/32, 44%). Three patients with 46,XX karyotype (3/18, 17%) presented between the ages of 1.5–2.5 years with Prader 4–5 virilization; two were diagnosed with SV-CAH, whereas one had 11-beta-hydroxylase deficiency. They underwent total hysterectomy and bilateral salpingo-oophorectomy between the ages of 4–7 years after confirmation of gender identity as males, and subsequently received pubertal hormone replacement therapy with T at puberty. As a result, 17 patients (17/32, 53%) were M^R^, and 15 (15/32, 47%) were F^R^.

The mean HC dose was 19.4 ± 4.4 mg/m^2^ per day before switching to Dex. ACTH level was > 100 pg/mL in 68% (15/22) of the patients. All patients had elevated adrenal androgen levels (Table 2). The daily fludrocortisone dose ranged from 0.025 to 0.1 mg in patients with SW-CAH. Mild hyponatremia was detected in two patients, and elevated renin levels in 55% (11/20) of the patients with SW-CAH due to treatment non-compliance. One patient with 11-beta-hydroxylase deficiency had hypernatremia, hypopotassemia, and hypertension, suggesting noncompliance with the treatment.

**Table 2:** Clinical and laboratory parameters of the patients at baseline and during follow-up

Variables			Normal values	F^R^ (*n* = 15)					M^R^ (*n* = 17)^a^				
				*n*	Baseline	*n*	Follow-up*	*p*	*n*	Baseline	*n*	Follow-up*	*p*
Tanner stage, n (%)		1 2 3 4 5		15	2 (13.3) 0 0 3 (20) 10 (66.7)	15	0 0 2 (13.3) 0 13 (86.7)	***0.003***	14	0 1 (7) 5 (35) 1 (7) 7 (49)	14	0 0 6 (43) 0 8 (57)	0.08
Overweight/obesity, n (%)				15	11 (73)	14	11 (78.5)	0.999	17	8 (47.1)	13	8 (61.5)	0.625
Testis volumes, mL^c^					-		-		14	20 (12)	14	25 (10)	***0.017***
Menstrual cycle (%)		Regular, n (%)		8^b^	2 (25)	7^b^	5 (71)	0.250		-		-	
Hirsutism, n (%)				15	7 (46)	15	4 (26.6)	0.500		-		-	
Blood pressure		Systolic, p^c^	< 95 p	11	61 (63)	13	67 (49)	0.906	15	56 (65)	12	73.5 (50)	0.959
		Diastolic, p^c^	< 95 p	11	74 (52)	13	73 (55.5)	0.259	15	80 (51)	12	89 (11.7)	0.499
Glucose (mg/dL)^d^			70–100	15	80.0 ± 7.3	13	80.1 ± 9.1	0.972	15	77.9 ± 7.2	14	76.8 ± 8.3	0.582
Sodium (mEq/L)^d^			136–146	13	138.1 ± 3.3	11	138.2 ± 2.8	0.789	15	138.7 ± 3.2	14	138.5 ± 2	0.823
Potassium (mEq/L)^d^			2.4–4.7	13	4.4 ± 0.2	11	4.5 ± 0.3	0.141	15	4.2 ± 0.4	14	4.4 ± 0.4	0.491
Renin (pg/mL)^c^			1.3–13.8	9	65.3 (47.6)	9	35 (99)	0.310	8	82.5 (186.2)	8	30.5 (16)	0.123
Gonadotropin and sex steroids	FSH (mIU/mL)^c^		M: 0.95–11.95 F: 1.5–33.4	11	3.6 (2.2)	6	4.8 (1.9)	0.500	10	1.7 (3.4)	8	4.3 (4)	0.075
	LH (mIU/mL)^c^		M: 0.57–12.07 F: 0.07–11.8	12	1.3 (2.6)	7	3.3 (1.2)	0.735	9	1.7 (2.3)	10	2.3 (1.9)	0.345
	Estradiol (pg/mL)^c^		11.8-175.6	12	46.9 (39.1)	7	36 (81.8)	0.612		-		-	
	Testosterone (ng/dL)^c^		M: 39–631 F: 7-11.8	13	71 (56.9)	11	13.8 (12.2)	***0.008***	15	309.0 (446.5)	12	425.9 (374)	0.790
Adrenal androgens	17OHP (ng/mL)^c^		0.6–2.15	15	38.2 (37.6)	12	2.7 (13.1)	***0.006***	13	47.7 (51.9)	12	2.3 (10)	***0.002***
	11DOC (ng/mL)^c^		< 7.2	5	5.3 (87.8)	4	3.5 (36.1)	0.655	8	38.5 (174.5)	7	0.8 (0.8)	***0.043***
	A4 (ng/mL)^c^		0.7–2.4	13	6.4 (6.7)	12	0.4 (0.8)	***0.005***	15	5.2 (7.9)	10	0.31 (0.4)	***0.005***
A4/T ratio			< 0.2		-		-		10	2.6 (3.5)	10	0.07 (0.07)	***0.005***
ACTH (pg/mL)^c^			0–46	9	231 (280.7)	7	22.6 (33.7)	***0.043***	13	564.0 (1.112)	9	6.2 (19.1)	***0.028***
Testicular / ovarian ART, n (%)				11	0	8	0		12	7 (58)	12	4 (25)	0.125

^a^Three M^R^ patients with 46,XX karyotype were not included in the analysis of Tanner stages, testis volumes, gonadotropin and sex steroid levels, A4/T ratio, and testicular ART analysis. ^b^Evaluated for the patients who had menarche or were over 16 years of age. ^c^median (IQR), ^d^mean±SD, *Data at 6–12 months. A4: 1,4-delta-androstenedione, A4/T ratio: 1,4-delta-androstenedione/testosterone ratio, ACTH: adrenocorticotropic hormone, ART: adrenal rest tumor, F^R^: female-reared, M^R^: male-reared, p: percentile, 11DOC: 11-deoxycortisole, 17OHP: 17-hydroxyprogesterone

The mean daily starting dose of Dex was 15.4 ± 5.4 mg/m^2^ of HC equivalent (0.6 ± 0.2 mg/day Dex; range 0.5-1.0 mg/day) and after dose adjustment, the mean dose decreased to 13.7 ± 6.5 mg/m^2^ (0.5 ± 0.2 mg/day Dex; range between 0.25 and 1.0 mg/day) based on the adrenal androgen levels on follow-up (*p* = 0.144). Only four patients (4/32, 12.5%) with initial doses of 0.375–0.75 mg/day Dex (8.7–17.9 mg/m^2^ of HC equivalent) required an escalation in Dex dosage. In addition, during follow-up, 72% of the patients (23/32) maintained normal androgen levels with a dose of ≤ 0.5 mg/day Dex (≤ 14 mg/m^2^ of HC equivalent). None of the patients exhibited symptoms/signs of adrenal insufficiency or adrenal crisis during follow-up.

**Fig. 1:**
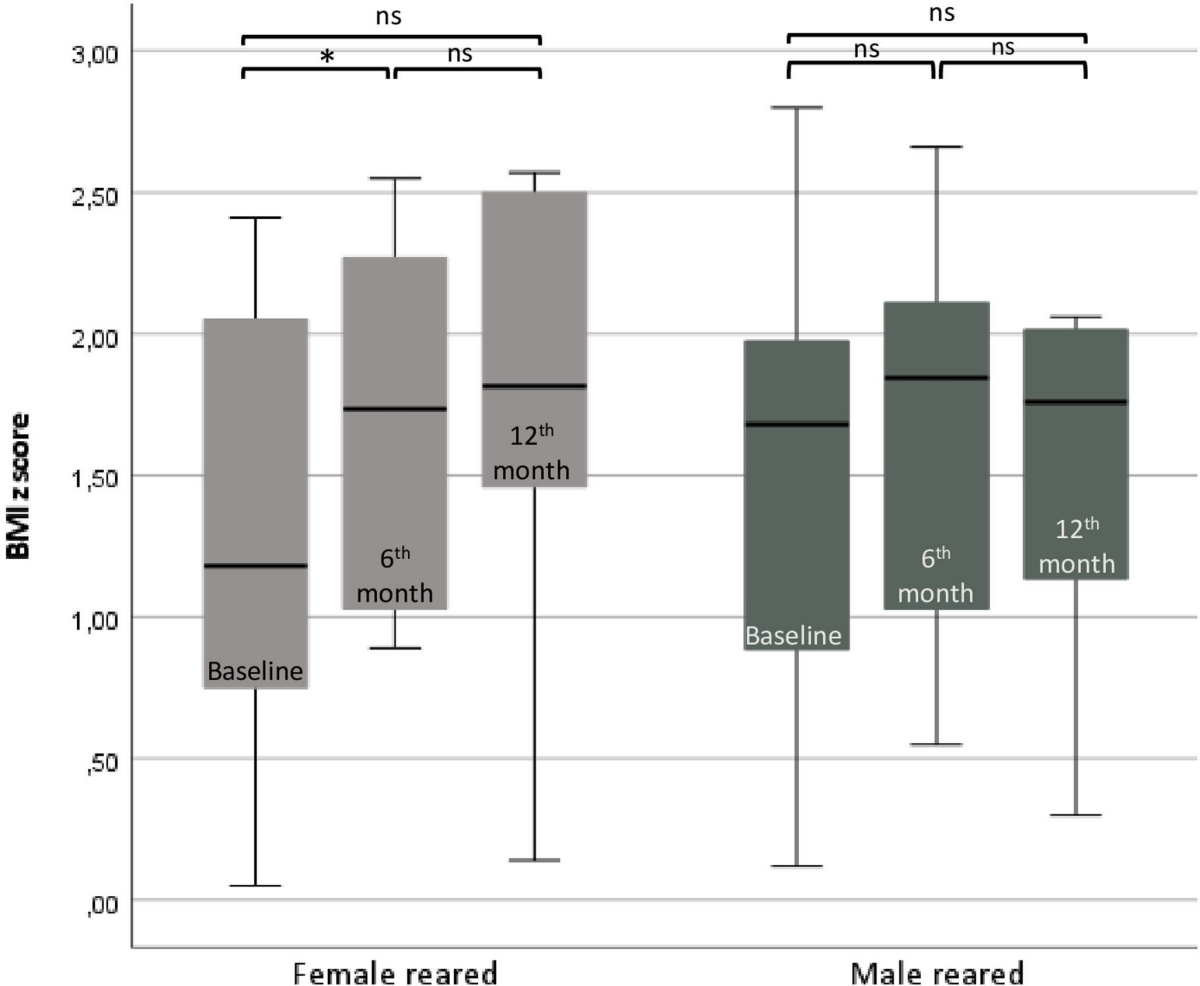
BMI z score over 12 months in female and male reared patients at baseline, 6th, and 12th months. Boxplots display the median (center line) and interquartile range (box); whiskers extend to 1.5×IQR. *p < 0.05, ns *p* ≥ 0.05; Wilcoxon signed-rank test. BMI: body mass index

The mean age of F^R^ patients (15/32, 47%) was 15.2 ± 2.3 years. The clinical characteristics of F^R^ patients before the treatment switch are given in Tables 1 and 2. Following the transition to Dex therapy, both 17OHP (*p* < 0.01) and A4 (*p* < 0.01) levels showed a significant decrease after 6–12 months (Table 2). Two patients (2/15, 12.5%) with delayed puberty advanced to T_B_3 puberty by the sixth-month follow-up. Among the patients with T_B_4 puberty (3/15, 20%), all progressed to T_B_5, and two of them experienced menarche. 46% of the patients (7/15) had hirsutism before the switch, and three of them (3/7, 42.8%) exhibited improvement on follow-up. One patient with 11-beta-hydroxylase deficiency who had regular menstrual cycles despite elevated androgen levels showed improvement in hirsutism, hypertension, hypernatremia, and hypopotassemia with the Dex therapy. Among the eight patients, initially, six of them had menstrual irregularity (6/8, 75%). Two patients (one with metrorrhagia and the other with oligomenorrhea) had regular menstruation afterward. One patient with oligomenorrhea was lost to follow-up after androgen levels normalized. One of the remaining three patients, aged 18.2 years, who had primary amenorrhea, subsequently developed menstrual cycles following the therapy switch. The last two patients with primary amenorrhea, aged 16 and 16.1 years, did not experience menarche during two years of Dex therapy, despite having normalized androgen and ACTH levels (Table 2). Their gonadotropin levels were normal, while E2 levels were low; ultrasound showed a thin endometrial lining in both patients. After the treatment switch, BMI z score increased significantly at the sixth month of therapy (*p* = 0.014), then remained similar at the twelfth month of therapy (*p* = 0.612) (Fig. 1). The prevalence of cases with overweight/obesity did not increase compared to the baseline (*p* > 0.999). The change in BMI z score at the sixth month was not correlated with either the cumulative GC dosage (*r* = 0.022, *p* = 0.943) or the starting dose of Dex (*r* = 0.077, *p* = 0.803). At follow-up, the prevalence of SBP and DBP ≥ 95th percentile did not change over time after Dex therapy (*p* > 0.999, *p* > 0.999, respectively) (Table 2). Five patients developed red-purple striae, and additionally, one patient developed acne and plethora.

**Fig. 2:**
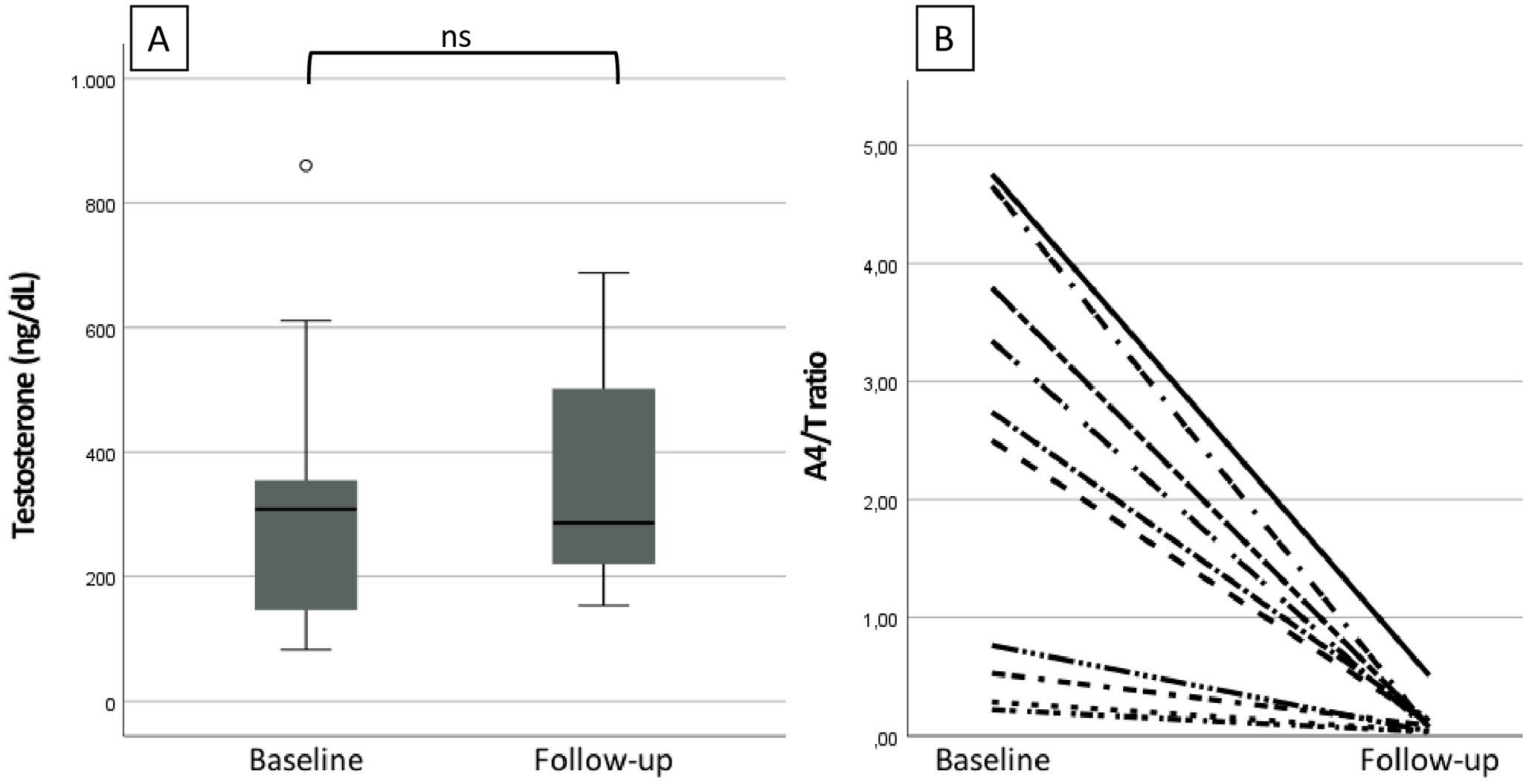
Changes in serum testosterone and A4/T ratio from baseline to follow-up of ten M^R^ patients with 46,XY karyotype. (**A**) Boxplots show serum testosterone (ng/dL) at baseline and follow-up; the center line indicates the median, the box the interquartile range (IQR), and whiskers extend to 1.5×IQR. The outlier is shown. ns *p* ≥ 0.05, Wilcoxon signed-rank test. (**B**) Individual trajectories of the A4/T ratio from baseline to follow-up (each line represents one patient). A4/T ratio: 1,4-delta-androstenedione/testosterone ratio

The mean age of the M^R^ patients (17/33, 52%) was 15.7 ± 1.7 years. The clinical characteristics of M^R^ patients before the treatment switch are given in Tables 1 and 2. Following the transition to Dex therapy, both 17OHP (*p* < 0.01) and A4 (*p* < 0.01) levels showed a significant decrease after 6–12 months (Table 2). Three M^R^ patients with 46,XX karyotype received pubertal hormone replacement therapy with T. At the time of switch, 14 M^R^ patients had pubertal development at Tanner stages 2–5, testes volumes (TV) were 25 mL in 43% (6/14) of the patients, and in the remaining patients (8/14), they ranged between 6 and 20 mL (median 12 (8)). After the switch, in a 6-12-month period, TV increased in all but one patient (7/8, 87.5%), and TV ranged between 12 and 25 mL (median 15 (10), *p* = 0.017). The median T measurement did not change statistically (*p* = 0.790). Ten patients with 46,XY M^R^ had simultaneous measurement of A4 and T levels. At the time of switch, the A4/T ratio was higher than 0.2 in all 10 M^R^ patients (100%), and in 60% (6/10) of them, the ratio exceeded 1. After the switch, A4/T ratio declined below 1 in all patients (10/10) (*p* = 0.005), and in 90% of them (9/10), the ratio fell below 0.2 (Fig. 2). Before the switch, 58% of the M^R^ patients (7/12) had TART on ultrasound examination. TART disappeared in three patients (3/7, 43%), and remained unchanged in the remaining patients within a one-year follow-up. After the treatment switch, BMI z score was similar at the sixth month (*p* = 0.325), and at the twelfth month of the therapy (*p* = 0.674) (Fig. 1). 61.5% (8/13) of the patients were overweight or obese at the sixth month of Dex treatment. Although the prevalence of cases with overweight/obesity increased, this change was not statistically significant (*p* = 0.625, compared to the baseline). At follow-up, the prevalence of SBP and DBP ≥ 95th percentile did not change over time after Dex therapy (*p* > 0.999, *p* > 0.999, respectively) (Table 2). Six patients developed red-purple striae at the 6th -12th month of Dex treatment.

## Discussion

Dexamethasone could be used to promote fertility by restoring the hypothalamo-pituitary-gonad axis in patients with CAH [32]. Using a similar approach, we replaced HC with Dex in patients with CAH who had reached final or near-final height and exhibited persistently elevated adrenal androgen levels, menstrual irregularities, pubertal delay, or TART despite receiving adequate dosages of HC. All patients experienced a reduction in adrenal androgen levels, and a significant proportion of patients demonstrated improved gonadal function, as evidenced by progression in pubertal stages, establishment of regular menstrual cycles, and preservation of gonadal T levels alongside decreased A4 levels.

Pubertal timing and progression are affected by the interaction of several factors, including genetic, internal, and external milieu. Hormonal imbalance associated with CAH may further complicate interactions during puberty. The onset of puberty may be early or delayed in patients with CAH [33–35], and pubertal progression appears to be even more complex. Völkl et al. reported that 98 patients with classic CAH experienced an early onset but a delayed progression of puberty in both genders, in comparison to the control group [36]. Due to early somatic growth and advanced bone age, gonadarche is expected to be earlier than the reference population [35]. Conversely, excessive adrenal androgens may interfere with the hypothalamo-pituitary-gonad axis, resulting in secondary gonadal failure, either directly or via conversion to estradiol through aromatization [37]. Thus, suppression of adrenal androgens is expected to resolve secondary hypogonadism [37]. In our cohort, two female patients with delayed puberty experienced pubertal progression subsequent to the suppression of adrenal androgens while on Dex. Hyperandrogenism can also cause anovulatory cycles and menstrual irregularities [38]. Menstrual irregularities were reported frequently, ranging from 33% to 64% of female patients [1, 2, 39]. In cohorts, spontaneous menarche was reported to occur in 60–80% of patients at a median age of 13 and 16 years, with a broad age range extending from 9 to 25 years [1, 39]. This implies that a substantial proportion experienced primary amenorrhea, highlighting its relatively high prevalence in this population. In our cohort, only 25% of patients had regular menstrual cycles; notably, these patients had persistent biochemical and clinical hyperandrogenemia, and regular cycles do not exclude anovulation. Three patients developed regular menstrual cycles following Dex treatment. Kaur et al. reported that among nine girls with regular bleeding, five were receiving Dex, and suggested that this treatment provided better compliance and hormonal control [39]. However, our two patients with SW-CAH did not experience menarche following Dex, even though their androgen levels returned to normal. Both patients exhibited normal gonadotropin levels, low E2, a thin endometrium, and small gonads. In the absence of androgen excess, adrenal progesterone production is suggested to remain high, resulting in menstrual irregularities in patients with CAH [40]. Persistently elevated progesterone levels can limit follicular growth, inhibit endometrial proliferation, and prevent endometrial shedding, leading to amenorrhea or oligomenorrhea. Further increase in GC dosage can suppress the production of adrenal progesterone, which may induce menstruation [40]. Hoimes-Walker et al. reported three patients with primary amenorrhea who had normal gonadotropin levels, reduced endometrial thickening, and non-suppressible serum progesterone levels despite suppression of 17OHP with Dex therapy; one of them experienced menarche only after bilateral adrenalectomy, suggesting that the progesterone is of adrenal origin [41]. It is unclear why adrenal progesterone remains elevated in the face of effective adrenal suppression. Findings in our patients may indeed suggest impaired folliculogenesis; however, we did not measure progesterone levels, and one should be cautious about increasing the GC dosage further, as it may lead to side effects. Combined estrogen-progesterone therapy can be the choice of treatment to achieve regular menstrual cycles in patients who do not desire fertility [42].

Maintaining gonadal function in male patients with CAH involves two primary issues. First, elevated adrenal androgen levels may suppress gonadotropins and result in secondary hypogonadism, similar to female patients [43]. Secondly, TART may adversely affect gonadal function by expanding and compressing normal testicular tissue, thereby hindering blood flow and promoting fibrosis within the testicular tissue [43]. In this case, alternative treatment approaches need to be implemented, such as increasing the GC dosage/frequency or adding long-acting GCs [44]. The efficacy of more frequent administration of HC is constrained due to non-compliance with treatment, which is associated with psychosocial changes induced by puberty. In our cohort, Dex therapy preserved T levels while reducing A4 levels, thereby reversing the A4/T ratio, suggesting a shift of T production from the adrenals to the testes, accompanied by an overall increase in TV. Schröder et al. reported a TART frequency of 38% among 188 male patients aged between 10 and 17 years [45]. TART frequency tends to increase with age and poor disease management [45, 46]. 58% of our patients with poor disease management had TART, and 43% of them achieved complete regression through more stringent disease control with Dex therapy.

Patients with CAH, especially females, demonstrate an earlier adiposity rebound and a high prevalence of early-onset obesity, independent of familial predisposition, suggesting that disease-related variables play a role in the development of obesity [1, 2, 47]. Furthermore, the association between the prevalence of obesity and higher doses/long-acting GCs suggests that obesity may also be treatment-related [1, 2]. Achieving a delicate balance between hypercortisolism and hyperandrogenism is challenging in a subset of patients with CAH. Our sample exhibiting inadequate disease control also had a high prevalence of obesity, suggesting the coexistence of hyperandrogenism and hypercortisolism, thus rendering it a challenging subset of patients to manage. In our study group, F^R^ patients had an increase in BMI after six months of Dex treatment, which subsequently stabilized; some patients also manifested Cushingoid features. While we were unable to establish a correlation between the initial and total cumulative dose of Dex, and weight gain, it is well-documented that there exists a significant relationship between GC dosage and weight gain [48]. Given that we were able to effectively decrease the dosage in a substantial proportion of patients, and the majority preserved normal androgen levels at doses of 0.5 mg/day Dex or lower, it can be recommended to commence Dex therapy with daily doses not exceeding 0.5 mg. Furthermore, dose adjustments should be made based on hormone assessments conducted promptly with short intervals, a maximum of 4 weeks. Given that sensitivity may vary among individuals, the dosage of GC should be tailored accordingly [49]. It is preferable to use Dex short-term at the lowest effective dose that prevents hyperandrogenemia, which minimizes side effects, until the patient demonstrates improved tolerance and compatibility with standard CAH management.

The current study has several limitations. First and foremost, the patient population is quite small, since our cohort comprises a distinct group of individuals with CAH, characterized by elevated androgen levels and gonadal dysfunction. Second, due to the absence of a universally accepted 17OHP target and the variation of thresholds across studies, our definition should be interpreted as an operational cut-off based on morning pre-dose sampling. Third, the retrospective design of this study may introduce bias regarding patient selection, non-homogeneous Dex dosages, and the documentation of missing laboratory data. Although all patient records contained the statement “compliance is good,” adherence had not been evaluated utilizing a standardized or validated methodology (e.g., structured questionnaires, pharmacy refill data, or pill counts). Consequently, a formal adherence outcome was not reported. Fourth, some patients were lost to follow-up. Fifth, the frequency of metabolic syndrome before and after Dex treatment could not be assessed due to the limited data available.

In conclusion, Dex, a potent long-acting GC, effectively suppresses adrenal androgens and could be employed in order to restore the hypothalamo-pituitary-gonad axis in children with CAH who have attained final or near-final height. To mitigate weight gain and metabolic adverse effects, treatment must be initiated with a low dosage, and androgen evaluations should be performed promptly.

## Data Availability

The datasets generated and analyzed in this study are not publicly available but are available from the corresponding author on reasonable request.
